# Whom should I rely on for my future care? Patterns of care expectations and intergenerational correlates among ageing Chinese adults in Hong Kong

**DOI:** 10.1111/hsc.12629

**Published:** 2018-08-14

**Authors:** Xue Bai

**Affiliations:** ^1^ Department of Applied Social Sciences The Hong Kong Polytechnic University Hong Kong China

**Keywords:** care expectations, Chinese, latent class analysis, old age and social care, older people

## Abstract

This study examined ageing parents' care expectations across multiple care domains (financial and material, emotional, personal and informational) towards filial and formal sources and identified intergenerational correlates of care expectation patterns using a proposed care expectation model. Data of 780 eligible ageing parents were drawn from a representative household survey of ageing adults (≥50 years) conducted in 2016–2017. Latent class analysis was used to examine the typological structure underlying ageing parents' care expectations. Four patterns of care expectations were discovered: mixed–maximal, filial–modest, formal–modest and neither–minimal. Multinomial logistic regression analysis was conducted to validate the newly proposed care expectation model. In addition to certain predisposing factors (participants' age, sex and education), parental enabling resources (economic status), health characteristics (physical, mental and functional health status), children‐related enabling characteristics (number of sons and marital status of children), and intergenerational enabling circumstances (intergenerational relationships and caregiving to their own parents) were introduced into the model and found to be associated with ageing parents' care expectations. The findings can inform policy and programmes that effectively respond to ageing adults' diverse care expectations in Hong Kong and have implications for other Asian societies facing rapid population ageing and increasing care demands.

**What is known about the topic**

- Filial care from adult children is becoming increasing unreliable for Chinese older adults in changing family and sociocultural contexts.
- Parental and children‐related characteristics influence older adults' beliefs about what the most appropriate forms and sources of care are when assistance and support are required.

**What this paper adds**

- The results of latent class analysis reveal that ageing Chinese parents in Hong Kong hold four patterns of care expectation of filial and formal caregivers across multiple care domains: mixed–maximal, filial–modest, formal–modest and neither–minimal.
- The care expectation model is developed, and the results of multinomial logistic regression analysis indicate that predisposing factors, parental enabling resources, health characteristics, children‐related enabling characteristics and intergenerational enabling circumstances jointly influence ageing parents' care expectation patterns.

## 1 INTRODUCTION

Rapid population ageing and increased life expectancy pose great challenges to care provision for older adults. In Chinese societies, including that of Hong Kong, people traditionally adhered strongly to the Confucian value of filial piety and regarded family as the primary source of elder care within extended family living arrangements (Chui, 2012). The emphasis on filial obligations plays an influential role in shaping older Chinese adults' filial care expectations. However, such traditional care arrangements are becoming increasingly unreliable because of the transformation of family structures, values and functions because of the processes of modernisation and Westernisation (Bai, 2016; Chow & Bai, 2011; Zhan, 2004). Declining birth rates and increased participation of women in the labour market have greatly threatened the basis of informal care (Census & Statistics Department, 2014); the rising cost of living, globalisation and geographical mobility have undermined the capacity of the young generation in Hong Kong to take adequate care of their older parents (Bai, 2018). In response to new sociocultural realities, older adults in Hong Kong tend to modify their long‐lasting filial care expectations and develop more diverse care expectation patterns.

Care expectation refers to anticipation and beliefs about what the most appropriate forms and sources of care are when assistance and support are required in multiple care domains (e.g., financial and material, emotional, personal and informational) in the future. According to proactive coping theory (Aspinwall & Taylor, 1997), preparation for diverse care needs by formulating concrete care plans is a form of proactive coping to alleviate the potential negative effects of emerging care needs as stressors. A detailed understanding of older adults' care expectations could inform the allocation of resources, and development of policy and interventions intended to alleviate potential threats to future care arrangements and enhance the quality of care and family services.

In Hong Kong, more than 70% of community‐dwelling adults aged 60 years or over have chronic diseases, and approximately 25% require care and assistance in their daily living (Census & Statistics Department, 2009). However, older adults' views are seldom heard in regard to their expected care domains and sources. To fill this critical knowledge gap, the present study examined ageing parents' care expectations regarding filial and formal sources across varied care domains and identified influential correlates using a newly proposed care expectation model.

### 1.1 Sources and aspects of elder care and care expectation patterns

Financial and material care, emotional care, personal care and informational care are the common aspects of elder care and can be provided by informal sources (e.g., family, relatives, friends or neighbours) and formal sources (e.g., government, assisted living or nursing facilities or professionals). Financial and material care involves direct cash transfer, monthly bill payment, and shared family expenditure and medical expenses (Wangmo, 2010). Emotional care refers to companionship, respect, encouragement, comfort, trust, moral support, and the reduction or prevention of loneliness, fear, and isolation through regular contact, contact with other people, and activity participation (Lin, Li, Ji, & Wu, 2015; Wangmo, 2010). Personal care involves assistance with instrumental activities of daily living, such as meal preparation, taking of medication and housekeeping (Jacobs, Broese, van Groenou, de Boer, & Deeg, 2014). Informational care is increasingly crucial, with emphasis placed on providing easy access to information that empowers older people to remain healthy, make informed choices and exert control over their lives (Godfrey & Johnson, 2009).

The hierarchical compensatory model (Cantor, 1991) and task‐specific or supplementary model (Litwak, 1985) are two key models that attempt to explain older adults' preferences for care sources (Pinquart & Sörensen, 2002). Cantor's (1991) hierarchical compensatory model assumes that older adults' caregiver preferences are based on the closeness of their relations, from spouse and children to other relatives, friends or neighbours, and finally formal institutions. Litwak's (1985) task‐specific model suggests that informal and formal care coexist and supplement each other, with different caregivers suited to different care tasks. These perspectives served as the main theoretical underpinnings for our key hypothesis that older people may display differing care expectation patterns.

### 1.2 Determinants of older adults' care expectations: the missing role of intergenerational relations

Studies have identified a range of predictive factors of older adults' care expectations, including age (Lee, Netzer, & Coward, 1995), sex (Blieszner & Mancini, 1987), marital status (Suitor, Gilligan, Johnson, & Pillemer, 2014), education level (Lee et al., 1995), financial status (Zhan, Liu, & Guan, 2006) and self‐rated health status (Diwan, Lee, & Sen, 2011). However, most studies have focused only on individual characteristics, overlooking intergenerational factors. According to family systems theory (Bowen, 1966; Rosen, 1990), a family should be perceived as a complex entity consisting of interdependent and interacting family members and relationships. Older people's care expectations may be affected by family relational context, especially intergenerational circumstances.

Intergenerational relations are crucial for care planning and arrangements (Boerner, Carr, & Moorman, 2013; Watt et al., 2014). According to solidarity and conflict models (Bengtson & Roberts, 1991; Clarke, Preston, Raksin, & Bengtson, 1999), intergenerational relationships not only emphasise positive qualities of intergenerational bonds but comprise negative aspects. A four‐factor (i.e., consensual–normative solidarity, structural–associational solidarity, affectual closeness and intergenerational conflict) model was validated in a study of older adults in Hong Kong to conceptualise intergenerational relations (Bai, 2017). In addition, children's marital status and socioeconomic status may influence older people's care expectations since in China, daughters‐in‐law are expected sources of support for elder care (Silverstein & Giarrusso, 2010), and older people may be more likely to expect children's care if their children are in good living conditions. Therefore, in the present study, the four dimensions of intergenerational relationships and parental and children‐related characteristics were for the first time jointly examined as predictors of care expectations.

### 1.3 Conceptual framework

**Figure 1:**
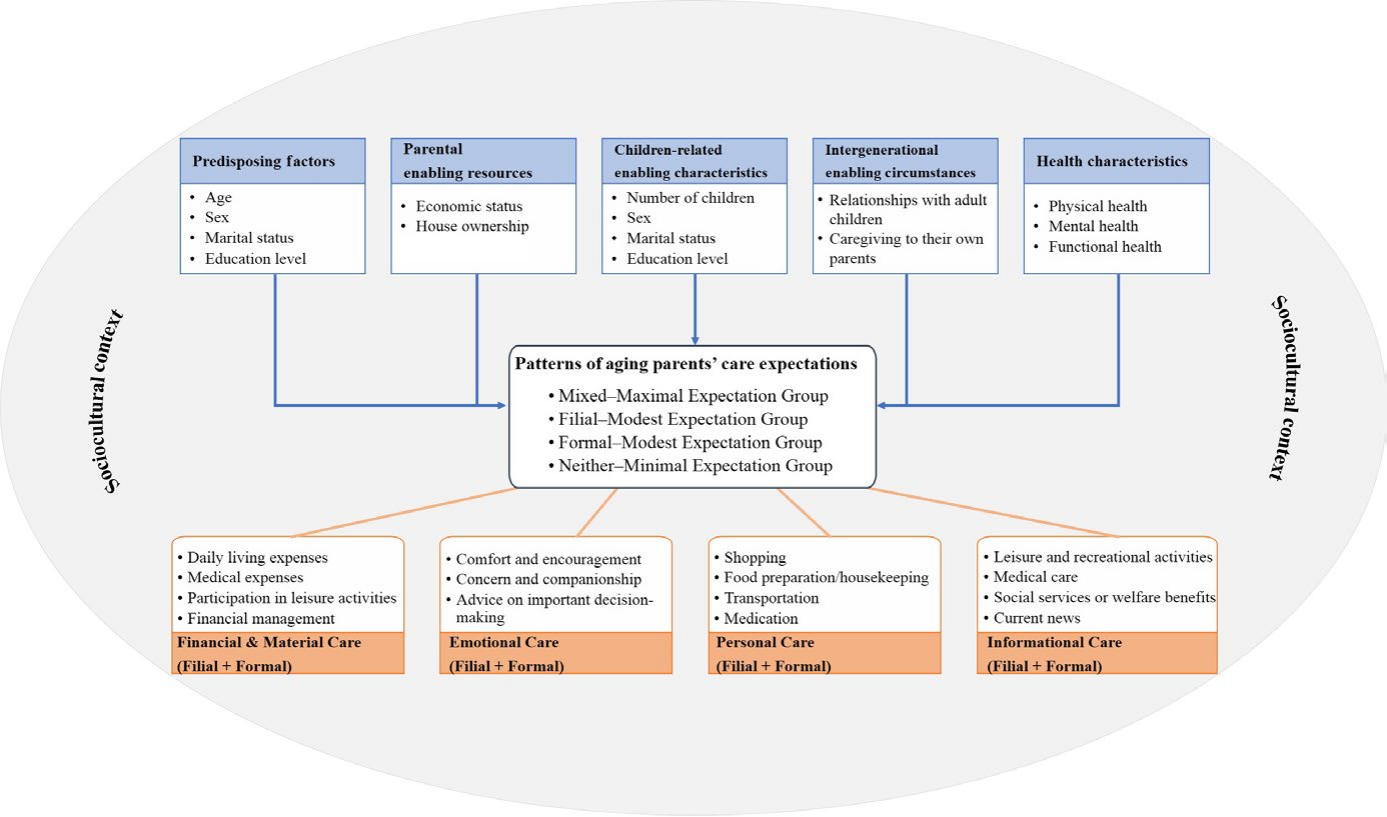
Proposed care expectation model

Andersen's (1995) healthcare utilisation model differentiated between predisposing characteristics, enabling resources, and health characteristics that affect healthcare utilisation. This model has since been widely used in long‐term care studies among diverse community‐ and institution‐dwelling ageing populations (Fu, Guo, Bai, & Chui, 2017; Slobbe, Wong, Verheij, Oers, & Polder, 2017). With reference to the healthcare utilisation model, the solidarity–conflict approach to understanding intergenerational relationships, critical theoretical perspectives about care provision and preferences, and existing empirical findings, a care expectation model was developed and used as a conceptual framework to guide the present study (Figure 1). Ageing parents' care expectation patterns were identified to determine their expectations of both their adult children and formal caregivers to provide financial and material, emotional, personal and informational care. Correlates of care expectation patterns were categorised into (a) predisposing characteristics, (b) parental enabling resources, (c) children‐related enabling characteristics, (d) intergenerational enabling circumstances and (e) health characteristics.

## 2 METHODS

### 2.1 Sampling

“Intergenerational Relationships and Care Expectations Among Chinese Older Adults in Hong Kong” is a representative household survey of 1,001 community‐dwelling ageing adults (a) aged 50 years or older, (b) residing in Hong Kong and (c) fluent in Cantonese or Mandarin. The data in the present study were drawn from this research project, which employed a two‐stage stratified sampling strategy. The sampling frame maintained by the Census and Statistics Department was first stratified by geographical area, and the type of quarter before 5,000 addresses was randomly selected using the systematic replicated sampling technique. For each selected household, if more than one older adult was eligible for the study, one was chosen using the earliest birthday method. After invalid addresses and addresses where no eligible person resided were excluded, a final sample of 1,001 participants was achieved.

### 2.2 Data collection

Trained interviewers conducted face‐to‐face household interviews from November 2016 to March 2017. Computer‐assisted personal interviews were used for data collection. Notification letters were sent to the selected households prior to visits. During the visits, out of the 1,966 valid participants, 234 refused to participate in the survey, and 731 were not contactable after more than five visits at various times of the day and on different days of the week. The remaining 1,001 participants completed the questionnaire, which lasted approximately 40 minutes, yielding a response rate of 50.9%. Ethical approval was obtained from the Human Subject Ethics Subcommittee of the author's affiliated university.

### 2.3 Measures

#### 2.3.1 Care expectations

Participants' expectations of filial and formal care were measured separately for financial and material, emotional, personal and informational care, using a 5‐point Likert scale (1 = no expectation; 5 = very high expectation). Concerning financial and material care, participants rated the extent to which they expected to receive financial and material assistance for their (a) daily expenses, (b) medical expenses, (c) participation in leisure activities and (d) assistance in investment and financial management from children and formal sources. For emotional care, participants rated the extent to which they expected to receive (a) comfort and encouragement, (b) concern and companionship, and (c) advice on making major decisions from children and formal sources. Personal care expectations were measured by asking the participants to rate the extent to which they expected to receive support in (a) daily activities, (b) housekeeping and (c) health monitoring from their adult children and formal caregivers. Informational care expectations were measured by the extent to which participants expected their adult children and formal sources to provide information on (a) leisure and recreational activities, (b) medical care, (c) social services or welfare benefits and (d) current news. Cronbach's alphas of the measures of the four care domains from children were 0.93, 0.91, 0.84 and 0.96, respectively, and from formal sources were 0.77, 0.95, 0.84 and 0.928, respectively.

#### 2.3.2 Predisposing factors

Participants reported their age, sex (0 = female; 1 = male), marital status (0 = married; 1 = unmarried, including divorced or separated, widowed and never married), and years of education.

#### 2.3.3 Parental enabling resources

Parental enabling resources were assessed by participants' economic conditions and house ownership. Participants rated their economic condition from 1 (very poor) to 5 (very rich). House ownership status was dichotomised (0 = private property owners; 1 = nonowners).

#### 2.3.4 Children‐related enabling characteristics

Participants reported their children's sex, marital status, education level and income level. Using this information on sex, each participant's number of sons and daughters was calculated. Based on the marital information, participants were categorised (0 = at least one married child; 1 = no married children). Based on the education information, the education level of participants' children was coded (0 = having at least one child with college education or higher; 1 = having no such children).

#### 2.3.5 Intergenerational enabling circumstances

Participants' relationships with adult children and caregiving for their own parents served as intergenerational enabling characteristics. Bai's (2017) 13‐item Intergenerational Relationship Quality Scale for Aging Parents was used to measure participants' relationships with each of their adult children. This scale (Cronbach's alpha = 0.79) captured four domains of parent–adult children relationships: (a) structural–associational solidarity (e.g., “How often have you had face‐to‐face contact in the past 12 months?”), (b) affectual closeness (e.g., “What are your general feelings of closeness to him/her?”), (c) consensual–normative solidarity (e.g., “Overall, how similar are your opinions?”) and (d) intergenerational conflict (e.g., “How often do you have tense and strained feelings toward him/her?”). Participants rated all items using a 5‐point Likert scale, with a higher score indicating more favourable intergenerational relationship quality. The total score ranged from 13 to 65. Participants' overall relationship quality with their children across different domains was obtained by dividing each participant's total score by their number of adult children. Additionally, the participants were asked whether they provided or had provided (if their parents had passed away) financial, emotional and personal support to either or both of their own parents (0 = no; 1 = yes). The scores of the three items were added to represent the number of care domains that they provided to their parents.

#### 2.3.6 Health characteristics

Participants' physical, mental and functional health statuses were assessed. Physical health status was assessed using the number of diagnosed chronic diseases. Mental health status was evaluated on the basis of depressive symptoms assessed using the 5‐item Geriatric Depression Scale (GDS; Hoyl et al., 1999). The total scores ranged from 0 to 5, with a higher score indicating a higher level of depressive symptoms. The Cronbach's alpha of the GDS was 0.74 in the current sample.

Functional health was assessed using the 6‐item Washington Group (WG) General Disability Measure. The WG measure assesses difficulties in seeing, hearing, walking or climbing steps, remembering or concentrating, washing all over or dressing, and communicating (1 = no difficulty; 4 = unable). The total scores ranged from 6 to 24, with a higher score indicating a more severe disability. The WG measure is recommended by the United Nations Population Division and widely used in censuses and surveys internationally (Palmer & Harley, 2012). The scale had satisfactory internal consistency (Cronbach's alpha = 0.91) in the study sample.

### 2.4 Data analysis

SPSS 20.0 and Mplus 7.0 were used for data analysis. After excluding 185 adults without adult children and 36 cases with substantial missing data in the focal independent and dependent variables, 780 eligible cases were retained for final analysis. Descriptive analyses were first conducted to determine the main characteristics of the participants. Care expectation scores of different care domains and care sources were standardised for comparison purposes. The differences between the expectation scores towards children and formal caregivers and in the four care domains were calculated and examined. The participants were subsequently categorised into high‐ and low‐expectation groups from filial and formal sources in each domain of care using the median scores as cutoff points. Based on these results, latent class analysis (LCA), a person‐centric analytic tool was used to identify homogenous subgroups possessing unique patterns of care expectations towards filial and formal care sources. LCA is performed by computing a model with a single latent class and successively adding classes, checking for model fit and significance. The optimal numbers of the latent classes were determined mainly with reference to Bayesian information criterion (BIC), Lo–Mendell–Rubin likelihood ratio test (LMR‐LRT) and entropy. Low BICs, significant *p* values for the LMR‐LRT and entropy ≥ 0.80 indicate good fit (Nylund, Asparouhov, & Muthén, 2007). Last, multinomial logistic regression analysis was conducted to identify the factors that differentiate ageing parents' patterns of future care expectations, thereby validating the proposed care expectation model.

## 3 RESULTS

### 3.1 Description of sample

Table 1 reports the descriptive statistics on the characteristics of the study sample. The mean age of the participants was 68.72 years. Of the 780 participants, 43.5% were men, and 61.9% were married. The average participant had 6.33 years of education and rated the economic status as fair to poor. Only one‐fifth owned private property. The mean (standard deviation [*SD*]) numbers of adult sons and daughters were 1.28 (0.95) and 1.25 (1.11), respectively. 65.9% of the participants had at least one married child, and 20.9% had at least one child who had received college education or higher. The mean (*SD*) scores for the four domains of intergenerational relationships were 11.01 (2.27; affectual closeness), 8.45 (2.38; consensual–associational solidarity), 13.14 (3.65; structural–associational solidarity) and 11.99 (2.40; intergenerational conflict), respectively. On average, participants provided or had provided 1.73 (out of 3) domains of care for their own parents. Participants had a mean (*SD*) of 1.08 (1.31) types of chronic diseases. Additionally, they had mean (*SD*) scores of 1.06 (1.39) and 9.77 (4.03) for depressive symptoms and disability, respectively.

**Table 1:** Participant characteristics (*n* = 780)

Study variables	Mean (*SD*)/frequency (%)	Score range
Predisposing factors		
Age	68.72 (10.76)	50–102
Sex (male)	339 (43.5%)	[0, 1]
Marital status (married)	483 (61.9%)	[0, 1]
Education (in years)	6.33 (4.04)	0–18
Parental enabling resources		
Self‐perceived economic status	2.93 (0.59)	1–5
House ownership (private property owners)	159 (20.4%)	[0, 1]
Children‐related enabling characteristics		
Number of sons	1.28 (0.95)	0–5
Number of daughters	1.25 (1.11)	0–7
Having at least one married child	514 (65.9%)	[0, 1]
Having at least one child with college education	163 (20.9%)	[0, 1]
Intergenerational enabling circumstances		
Affectual closeness	11.01 (2.27)	3–15
Consensual–normative solidarity	8.45 (2.38)	3–15
Structural–associational solidarity	13.14 (3.65)	4–20
Intergenerational conflict	11.99 (2.40)	4–15
Caregiving to their own parents	1.73 (1.33)	0–3
Health characteristics		
Number of chronic diseases	1.08 (1.31)	0–6
Depressive symptoms	1.06 (1.39)	0–5
Disability	9.77 (4.03)	6–24
Filial: overall care expectations	2.96 (1.06)	1–5
Filial: financial and material care	2.94 (1.24)	1–5
Filial: emotional care	3.55 (1.14)	1–5
Filial: personal care	2.87 (1.20)	1–5
Filial: informational care	2.61 (1.37)	1–5
Formal: overall care expectations	2.00 (1.05)	1–5
Formal: financial and material care	2.54 (1.20)	1–5
Formal: emotional care	1.52 (1.11)	1–5
Formal: personal care	1.74 (1.18)	1–5
Formal: informational care	2.01 (1.37)	1–5
Filial − Formal: overall care expectations	0.96 (1.14)	−4 to 4
Financial and material care	0.40 (1.58)	−4 to 4
Emotional care	2.03 (1.43)	−4 to 4
Personal care	1.13 (1.39)	−4 to 4
Informational care	0.60 (1.34)	−4 to 4
Filial + Formal: overall care expectations	4.96 (1.78)	2–10
Financial and material care	5.48 (1.86)	2–10
Emotional care	5.08 (1.74)	2–10
Personal care	4.62 (1.93)	2–10
Informational care	4.62 (2.38)	2–10

Participants typically had higher care expectations of their children (M [filial] = 2.96, *SD* = 1.06) than of formal caregivers (M [formal] = 2.00, *SD* = 1.05). In addition, the difference between the expectations of children and formal caregivers was the smallest in the aspects of financial and material care (M [filial − formal] = 0.40, *SD* = 1.58), followed by informational care (M [filial − formal] = 0.60, *SD* = 1.34) and personal care (M [filial − formal] = 1.13, *SD* = 1.39), with the greatest mean difference observed for emotional care (M [filial − formal] = 2.03, *SD* = 1.43). The results further indicated that older parents generally had the highest expectations for financial and material care (M [filial + formal] = 5.48, *SD* = 1.86), followed by emotional care (M [filial + formal] = 5.08, *SD* = 1.74), personal care (M [filial + formal] = 4.62, *SD* = 1.93) and informational care (M [filial + formal] = 4.62, *SD* = 2.38).

### 3.2 Typology of ageing parents' care expectations

LCA was used to estimate care expectation patterns by categorising individuals. Table 2 presents the goodness‐of‐fit statistics for the five latent class models. The four‐class model had the lowest BIC. The LMR‐LRT *p* value indicated that the four‐class model was superior to models with three or fewer classes (Nylund et al., 2007). Although the LMR‐LRT *p*‐value was still significant (*p* = 0.02) for the five‐class model, but considering its higher BIC and more unfavourable concordance with theoretical justification and interpretability of the classes compared with the four‐class model, the four‐class model was employed.

**Table 2:** Fit indices for latent class models with 1–5 classes

Fit statistic	Number of classes				
	1	2	3	4	5
No. of free parameters	8	17	26	35	44
Log‐likelihood	−4,183	−3,661	−3,490	−3,396	−3,376
BIC	8,419	7,436	7,153	7,025	7,044
LMR‐LRT	N/A	1,026	337	185	40
LMR‐LRT, *p*‐value	N/A	0.000	0.000	0.000	0.022
Entropy	N/A	0.83	0.80	0.80	0.81

Note

BIC: Bayesian information criteria; LMR‐LRT: Lo–Mendell–Rubin likelihood ratio test.

**Figure 2:**
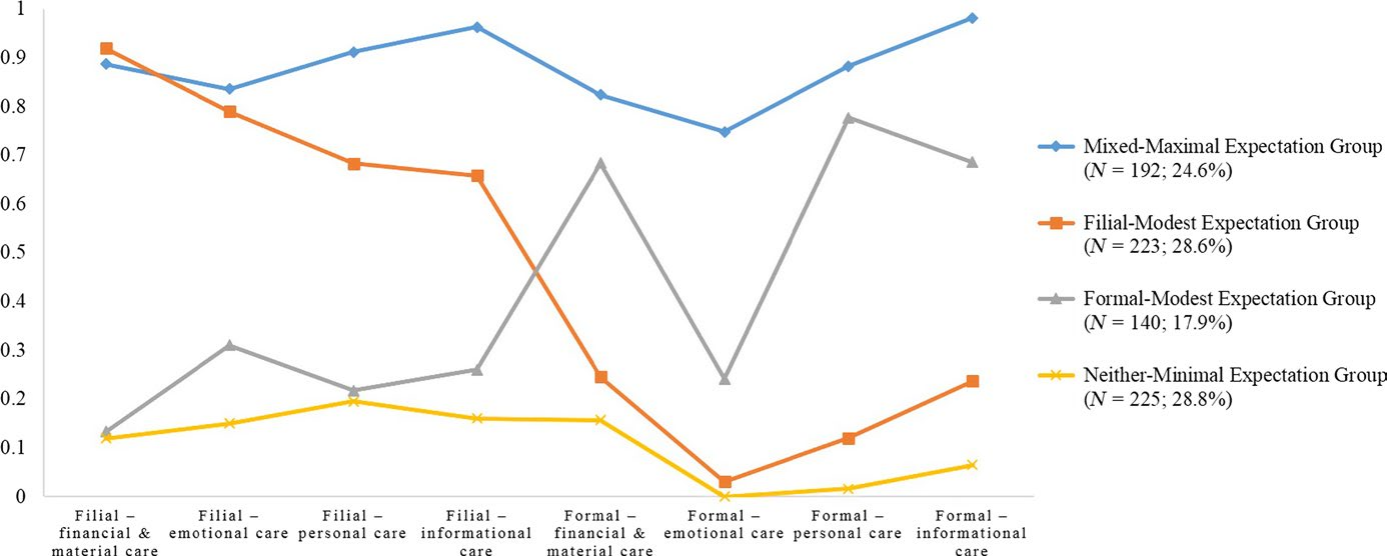
Probabilities of filial and formal care expectations by latent class assignment

Figure 2 illustrates the prevalence of cases in each class and the predicted probability of specific care domains conditioned on latent class assignment. Class 1 contained the 24.6% (*n = *192) of participants who had the highest probability of expecting care from both filial and formal sources. Thus, this class was labelled the “mixed–maximal expectation group” (mixed–maximal). Class 2 contained the approximately 28.6% (*n* = 223) of participants who had a relatively high probability of expecting filial care but a lower probability of expecting formal care. This class was labelled the “filial–modest expectation group” (filial–modest). Class 3 contained the approximately 17.9% (*n* = 140) of participants who had a relatively high probability of expecting formal care but a lower probability of expecting filial care. Accordingly, this class was labelled the “formal–modest expectation group” (formal–modest). Class 4 contained the approximately 28.8% (*n* = 225) of participants who were characterised as having the lowest probability of expecting filial and formal care. Thus, this class was labelled the “neither–minimal expectation group” (neither–minimal).

### 3.3 Testing the care expectation model to identify correlates of care expectation patterns

Table 3 reports the results obtained through multinomial logistic regression analysis, with the neither–minimal group used as the reference group for comparison with the other three groups. The significant correlates were also identified in the comparison between the filial–modest and the formal–modest groups. Compared with the neither–minimal group (Class 4), the following independent variables were associated with a higher likelihood of being in the mixed–maximal group: older age (odds ratio [OR]: 1.05, 95% confidence interval [CI]: [1.02, 1.08], *p* < 0.01), having at least one married child (OR: 3.44, 95% CI: [1.90, 6.23], *p* < 0.001), higher consensual–normative solidarity with adult children (OR: 1.59, 95% CI: [1.41, 1.78], *p* < 0.001), and providing more care to their own parents (OR: 1.28, 95% CI: [1.08, 1.53], *p* < 0.01). By contrast, more years of education (OR: 0.93, 95% CI: [0.87, 1.00], *p* < 0.1), having more sons (OR: 0.65, 95% CI: [0.50, 0.86], *p* < 0.01), less intergenerational conflict (OR: 0.77, 95% CI: [0.70, 0.86], *p* < 0.001), more chronic diseases (OR: 0.77, 95% CI: [0.61, 0.97], *p* < 0.05) and more severe disability (OR: 0.91, 95% CI: [0.85, 0.97], *p* < 0.01) indicated a lower likelihood of being in the mixed–maximal group.

**Table 3:** Multinomial logistic regression: correlates of care expectation patterns

*n* = 780	Mixed–maximal		Filial–modest		Formal–modest	
	OR	95% CI	OR	95% CI	OR	95% CI
Predisposing factors						
Age in years	1.05**	1.02–1.08	1.05**	1.02–1.08	1.03*	1.00–1.07
Female (reference is male)	1.19	0.71–1.99	2.03**	1.25–3.29	1.42	0.82–2.44
Married (reference is not married)	0.76	0.45–1.29	0.72	0.45–1.16	0.83	0.49–1.42
Education (in years)	0.93^	0.87–1.00	0.94^	0.88–1.00	0.96	0.90–1.04
Parental enabling resources						
Self‐perceived economic status	0.73	0.47–1.13	1.42^	0.94–2.14	0.92	0.59–1.45
House owner (reference is tenant)	0.90	0.50–1.63	1.06	0.62–1.81	0.87	0.45–1.65
Children‐related enabling characteristics						
Number of sons	0.65**	0.50–0.86	0.80^	0.63–1.02	0.74*	0.56–0.98
Number of daughters	0.93	0.74–1.16	0.96	0.78–1.17	1.00	0.79–1.25
With at least one married child (reference is none)	3.44***	1.90–6.23	1.63^	0.96–2.75	0.87	0.49–1.56
With at least one college education child (reference is none)	1.59	0.91–2.78	1.08	0.64–1.82	1.35	0.75–2.41
Intergenerational enabling circumstances						
Affectual closeness	0.93	0.81–1.06	0.92	0.82–1.03	0.85*	0.75–0.97
Consensual‐normative solidarity	1.59***	1.41–1.78	1.40***	1.26–1.55	1.20**	1.07–1.35
Structural‐associational solidarity	1.06	0.97–1.15	1.07^	0.99–1.16	0.90*	0.83–0.98
Intergenerational conflict	0.77***	0.70–0.86	1.01	0.91–1.12	1.08	0.96–1.21
Caregiving to their own parents	1.28**	1.08–1.53	1.17^	1.00–1.38	1.14	0.95–1.37
Health characteristics						
Number of chronic diseases	0.77*	0.61–0.97	0.99	0.81–1.21	1.09	0.88–1.36
Depressive symptoms	1.00	0.81–1.24	0.90	0.74–1.10	1.16	0.95–1.41
Disability	0.91**	0.85–0.97	0.97	0.91–1.03	0.92*	0.86–0.99

^*^Reference group: neither–minimal; *p* < 0.05. ^**^ *p* < 0.01.^***^ *p* < 0.001.^^^ *p* < 0.1.

Some predisposing factors, children‐enabling characteristics and intergenerational enabling circumstances were significantly more associated with membership in the filial–modest group than the neither–minimal group. Specifically, participants who were older (OR: 1.05, 95% CI: [1.02, 1.08], *p* < 0.01), were women (OR: 2.03, 95% CI: [1.25, 3.29], *p* < 0.01), had higher self‐perceived economic status (OR: 1.42, 95% CI: [0.94, 2.14], *p* < 0.1), with at least one married child (OR:1.63, 95% CI: [0.96, 2.75], *p* < 0.1), with higher consensual–normative solidarity (OR: 1.40, 95% CI: [1.26, 1.55], *p* < 0.001) and structural–associational solidarity (OR: 1.07, 95% CI: [0.99, 1.16], *p* < 0.1) with their children, and who provided more care to their own parents (OR: 1.17, 95% CI: [1.00, 1.38], *p* < 0.1) were more likely to be in the filial–modest group. By contrast, more years of education (OR: 0.94, 95% CI: [0.88, 1.00], *p* < 0.1) and having more sons (OR: 0.80, 95% CI: [0.63, 1.02], *p* < 0.1) indicated a lower likelihood of being in the filial–modest group.

Older age (OR: 1.03, 95% CI: [1.00, 1.07], *p* < 0.01) and higher consensual–normative solidarity (OR: 1.20, 95% CI: [1.07, 1.35], *p* < 0.01) were significantly associated with an increased likelihood of being in the formal–modest group instead of the neither–minimal group. Participants who had more sons (OR: 0.74, 95% CI: [0.56, 0.98], *p* < 0.05), higher affectual closeness (OR: 0.85, 95% CI: [0.75, 0.97], *p* < 0.05) and structural–associational solidarity (OR: 0.90, 95% CI: [0.83, 0.98], *p* < 0.05) with their children, and more severe disability (OR: 0.92, 95% CI: [0.86, 0.99], *p* < 0.05) were more likely to be clustered into the neither–minimal group than into the formal–modest group.

None of the predisposing factors were significantly related to classification between the filial–modest and formal–modest groups. The participants who had a higher self‐perceived economic status, a parental enabling resource (OR: 1.54, 95% CI: [0.97, 2.44], *p* < 0.1), at least one married child, a children‐related enabling characteristic (OR: 1.86, 95% CI: [1.02, 3.41], *p* < 0.05), and higher consensual–normative solidarity (OR: 1.17, 95% CI: [1.04, 1.31], *p* < 0.05) and structural–associational solidarity (OR: 1.18, 95% CI: [1.08, 1.29], *p* < 0.001) with their children were more likely to be members of the filial–modest group. The presence of more depressive symptoms (OR: 0.78, 95% CI: [0.63, 0.96], *p* < 0.05) was significantly related to decreased likelihood of being in the filial–modest group relative to the formal–modest group.

The goodness of fit indicators were Pearson χ^2^ = 2,204.77 (*df* = 2,157, *p* = 0.23), Deviance χ^2^ = 1,715.12 (*df = *2,157, *p* = 1.00), Cox and Snell *R* ^2^ = 0.340 and Nagelkerke *R* ^2^ = 0.364. The Akaike information criterion (AIC) in the final model (AIC = 1,829.12) was lower than that with intercept only (AIC = 2,028.61). The favourable model fit was further supported by the results of the likelihood ratio test, with a χ^2^ of 307.49 (*df = *54, *p *< 0.001), indicating that the final model was more effective than an intercept‐only model. The overall correct prediction of the group membership was 48.0%, suggesting a substantial improvement over chance level. Correct predictions were most frequent for the neither–minimal group (57.9%), followed by the filial–modest group (49.5%), the mixed–maximal group (48.6%) and the formal–modest group (27.6%).

## 4 DISCUSSION AND IMPLICATIONS

To the best of our knowledge, this is the first study to systematically examine ageing parents' diverse care expectation patterns across multiple care domains towards filial and formal caregivers. LCA of a wide range of indicators of financial and material, emotional, personal and informational care yielded four classes of care expectations: mixed–maximal, filial–modest, formal–modest and neither–minimal expectations. This study developed and validated a comprehensive care expectation model to effectively unravel the correlates of ageing parents' care expectations. In addition to certain predisposing factors, parental enabling resources, health characteristics, children‐related enabling characteristics and intergenerational enabling circumstances were found to be significantly associated with care expectation patterns.

As demonstrated by the LCA results, ageing Chinese parents in Hong Kong exhibited four care expectation patterns. Consistent with Cantor's (1991) hierarchical compensatory model, the participants tended to prefer informal over formal caregivers, especially for emotional and personal care, whereas they had more balanced expectations towards filial and formal sources regarding financial, material and informational care. This finding is aligned with Litwak's (1985) task‐specific model, which suggests that informal or formal care may coexist and supplement each other but are suited for different care tasks. The results further revealed that ageing parents had the highest expectations of financial and material care, followed by emotional care, personal care and informational care. Therefore, care policy and services could consider prioritising older people's financial and informational care needs. A recent qualitative Photovoice study found that ageing adults in Hong Kong experienced substantial insecurity and fear regarding future finances and had strong expectations towards the government for financial protection in old age (Bai, 2018). Furthermore, retirement protection policies should change from a needs‐based to rights‐based approach to foster empowerment and satisfy older people's financial care needs and enable them to age with dignity.

Predisposing factors, including age and sex, were significantly related to care expectation patterns among ageing parents, whereas marital status was not. Those who held neither–minimal care expectations were more likely to be younger than those in other groups. Mothers were twice as likely as fathers to expect filial care. This is consistent with previous findings that women are more likely than men to mention children as their first source of support (Spitze & Ward, 2000), possibly because older mothers generally have a closer relationship with their children than fathers have.

Concerning parental enabling resources, inconsistent with previous findings (Pinquart & Sörensen, 2002), this study found that higher self‐perceived economic status was marginally significantly related to a higher likelihood of being in the filial–modest group rather than the formal–modest group or the neither–minimal group. According to social exchange theory (Cook & Emerson, 1987), people expect to receive fair returns for their expenditures; therefore, it is reasonable that parents of higher economic status are more likely to invest in their children and in turn expect more care from them.

The results further revealed that children‐related characteristics, such as having more sons, were significantly associated with a higher likelihood of expecting neither–minimal care relative to expecting mixed–maximal, filial–modest and formal–modest care; however, the number of daughters was not a significant determinant. This finding contrasts with the traditional care preference for sons in Chinese societies. This is possibly because of older people's ambivalent attitudes towards filial care. Although children are more expected to provide support than formal sources, older parents are unwilling to burden their children because the younger generation is currently under substantial pressure to support their own families. As proposed by Hanaoka and Norton (2008), time‐related opportunity costs are higher for sons than daughters for care provision. Moreover, older Chinese adults are expected to perceive having more sons a significant barrier to using formal care given the cultural value of *mianzi* (face). Therefore, this type of parent may expect neither formal nor filial care. Additionally, parents of married children were found to be more likely to have mixed–maximal or filial–modest than neither–minimal care expectations, and they were more likely to expect filial over formal care. This may be because the expanded availability of potential, with sons‐ and daughters‐in‐law also able to provide care, may influence older people's care expectations (Hanaoka & Norton, 2008).

Intergenerational enabling circumstances were found to be significantly associated with ageing parents' care expectation patterns. For instance, higher levels of structural–associational solidarity were associated with a slightly higher probability of expecting filial care at a marginally significant level (*p* < 0.1). Higher levels of consensual–normative solidarity were found to be related to a lower probability of holding neither–minimal care expectations. This is because in the current study, one of the items measuring consensual–normative solidarity assessed the similarity of opinions concerning filial responsibilities in elder care and indicated that the two generations had already discussed and agreed on caregiving so that the older parents would have relatively higher expectations of filial, formal or mixed‐maximal care. This finding provides additional evidence of the value of intergenerational relationships for Chinese older adults.

Notably, those who had mixed–maximal care expectations were more likely to have higher levels of conflict with their children. A possible explanation is that these older adults are more demanding parents than other parents and, therefore, face conflict with their adult children more frequently. Moreover, the higher the level of caregiving participants provided for their own parents, the more likely they were to have filial–modest or mixed–maximal care expectations. This may be because that being a caregiver themselves reflected their familial care values to some extent; additionally, their experiences of caregiving to their own parents may increase their awareness about future care needs and enhance their care expectations. Professionals and service providers must enhance awareness of intergenerational care planning in family contexts.

Health characteristics were found to be significantly associated with care expectation patterns; however, the directions of the effects were unexpected. Less favourable health status (i.e., more chronic diseases and higher levels of disability) was found to be associated with a lower likelihood of having mixed–maximal and formal–modest care expectations in relative to neither–minimal care expectations. A study reported that many Chinese older adults tended to perceive themselves as a burden on family and society (Bai, Lai, & Guo, 2016). Older people of unfavourable physical and functional conditions may be reluctant to burden their children, and their children's care may not completely meet their needs. Some may avoid considering problems such as future loss of independence and discussing future care plans with their family. These older adults may have had relatively low expectations of formal care because of the barriers and long waiting lists in Hong Kong to access subsidised and private residential care homes.

This study had several limitations. First, cross‐sectional data were used, which limited the ability to establish causal relationships. Quantitative studies with a longitudinal design and qualitative studies are required to further unravel and contextualise the complex relationships between older adults' intergenerational circumstances and care expectations. Second, the present study focused only on adult children as the source of informal care. Future studies could expand the model to cover other informal sources and more specific care types to inform tailored care and service provision. Characteristics of the children, such as their financial status and living conditions, could be further added into the model in future studies. Third, childless older adults were not included in the data analysis, because intergenerational enabling circumstances were employed as a focal group of predictors. Future studies could include this group of older adults who are more vulnerable and in need of various forms of care and assistance.

## 5 CONCLUSION

Despite the limitations, the care expectation model proposed in this study provided a framework to facilitate understanding of aspects of ageing adults' perspectives of intergenerational relations and multifaceted care expectations and identified crucial intergenerational correlates of different expectation patterns. The findings elucidate ageing adults' diverse care expectations of both informal and formal sources, identify the areas in which ageing adults expect care and support from their adult children and areas in which they expect support from formal sources and highlight the importance of intergenerational care planning in family contexts. The study findings may further inform the development of proactive policy and programme responses to future care expectations to ensure that various support sources can effectively respond to ageing adults' diverse care needs and preferences in Hong Kong. These findings may also have implications for other societies facing rapid population ageing and increasing care demands.
